# New Transferrin Receptor-Targeted Peptide–Doxorubicin Conjugates: Synthesis and In Vitro Antitumor Activity

**DOI:** 10.3390/molecules29081758

**Published:** 2024-04-12

**Authors:** Jiale Yu, Xiaoxia Mao, Xue Yang, Guiqin Zhao, Songtao Li

**Affiliations:** 1 Hebei Province Key Laboratory of Research and Development of Traditional Chinese Medicine, Institute of Chinese Mateia Medica, Chengde Medical University, Chengde 067000, China; 15102526322@163.com (J.Y.); mao0505@163.com (X.M.); zhaoguiqin1971@sina.com (G.Z.); 2 School of Basic Medical Sciences, Chengde Medical University, Chengde 067000, China; xyang_cdmu@163.com

**Keywords:** transferrin receptor, doxorubicin, ^D^T7, peptide–drug conjugates, targeted antitumor activity

## Abstract

Poor selectivity to tumor cells is a major drawback in the clinical application of the antitumor drug doxorubicin (DOX). Peptide–drug conjugates (PDCs) constructed by modifying antitumor drugs with peptide ligands that have high affinity to certain overexpressed receptors in tumor cells are increasingly assessed for their possibility of tumor-selective drug delivery. However, peptide ligands composed of natural L-configuration amino acids have the defects of easy enzymatic degradation and insufficient biological stability. In this study, two new PDCs (^L^T7-SS-DOX and ^D^T7-SS-DOX) were designed and synthesized by conjugating a transferrin receptor (TfR) peptide ligand ^L^T7 (HAIYPRH) and its retro-inverso analog ^D^T7 (hrpyiah), respectively, with DOX via a disulfide bond linker. Both conjugates exhibited targeted antiproliferative effects on TfR overexpressed tumor cells and little toxicity to TfR low-expressed normal cells compared with free DOX. Moreover, the ^D^T7-SS-DOX conjugate possessed higher serum stability, more sustained reduction-triggered drug release characteristics, and stronger in vitro antiproliferative activity as compared to ^L^T7-SS-DOX. In conclusion, the coupling of antitumor drugs with the ^D^T7 peptide ligand can be used as a promising strategy for the further development of stable and efficient PDCs with the potential to facilitate TfR-targeted drug delivery.

## 1. Introduction

The clinical application of most antitumor agents has been greatly restricted due to severe toxic and side effects resulting from the inherently poor selectivity to tumor cells [1]. So far, diversified strategies have been developed to improve the specificity of these agents to tumor cells. One of the most effective approaches to target the tumor site is constructing peptide–drug conjugates (PDCs) by coupling antitumor drugs with tumor-homing peptide ligands that have high affinity to the specific receptors overexpressed on tumor cells via a cleavable linker [2,3]. In recent years, peptide ligands have been increasingly used for conjugation with drugs as a kind of “magic bullet” due to their advantages of easier preparation and structural modification, higher tissue permeability, and lower immunogenicity compared with protein ligands such as monoclonal antibodies [4,5].

Transferrin receptor (TfR) is an important transmembrane glycoprotein that plays a critical role in cellular uptake of iron by interacting with its natural ligand transferrin (Tf) [6]. Because of the rapid proliferation of tumor cells and increased demand for iron, TfR has been proven to be overexpressed in various kinds of tumor cells, including brain, liver, and lung cancers, whereas it is low-expressed in normal cells [7,8], which makes it an excellent target for tumor-targeted drug delivery systems (DDSs). By conjugating drugs or modifying nano-drug carriers with TfR peptide ligands, it is expected to improve the selective antitumor effect of drugs on TfR overexpressed tumor cells [9,10,11].

Doxorubicin (DOX) is a widely used chemotherapeutical drug that kills tumor cells by inhibiting DNA topoisomerase II [12]. It has been verified that DOX is highly effective on various malignant tumors, including brain, liver, and lung cancers [13,14], which makes it one of the most commonly used models of antineoplastic drugs for constructing tumor-targeted DDS [15,16]. ^L^T7 (HAIYPRH) is a TfR homing peptide screened from the 7-mer phage display library, and its binding site for TfR is distinct from that of Tf [17], which can help to avoid competitive inhibition by endogenous Tf [18]. The N-terminus L-cysteine (Cys)-modified analog of ^L^T7, namely Cys-^L^T7 (CHAIYPRH), has been extensively used to construct TfR-targeted DDSs [19,20,21]. However, like most peptide ligands composed of natural L-configuration amino acids, ^L^T7 is also susceptible to proteolytic enzymes, leading to poor biostability and insufficient targeting potential in vivo. Recently, an analog of ^L^T7 which was designed by using the retro-inverso strategy, namely ^D^T7 (hrpyiah), has been proven to possess higher TfR affinity and serum stability in comparison with that of ^L^T7 [22]. Meanwhile, studies showed that the ^D^T7 peptide with a D-cysteine on C-terminus (^D^T7-Cys, hrpyiahc)-modified nanoparticles could be used as effective vectors for TfR-targeted drug delivery [23,24,25]. Nevertheless, the application of both Cys-^L^T7 and ^D^T7-Cys as the drug carrier for constructing PDCs is rarely reported now.

In this study, Cys-^L^T7 and ^D^T7-Cys were, respectively, connected with DOX via N-succinimidyl 3-(2-pyridyldithio) propionate (SPDP) as the cross-linker to afford two new PDCs (^L^T7-SS-DOX and ^D^T7-SS-DOX) containing an intramolecular disulfide bond. The in vitro serum stability, reduction-responsive drug release profile, and TfR-targeted antitumor activity of the two peptide–DOX conjugates were evaluated.

## 2. Results and Discussion

### 2.1. Synthesis of PDCs

**Scheme 1:**
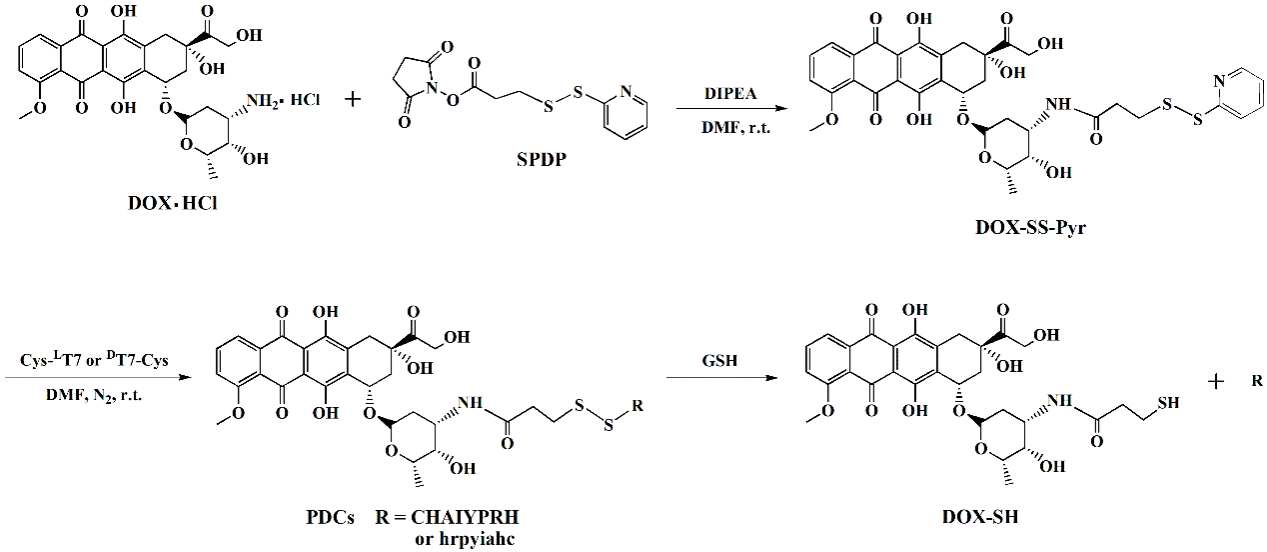
Synthetic route and reduction-triggered drug release of PDCs.

The ^L^T7-SS-DOX and ^D^T7-SS-DOX conjugates were synthesized in two steps, as shown in Scheme 1. First, the reaction between DOX and SPDP afforded DOX-SS-Pyr as a red solid (34.8 mg, 84.9% yield) with 95.3% purity; ESI MS (*m*/*z*) [C_35_H_36_N_2_O_12_S_2_]: calculated, 740.2; found, 741.1 [M + H]^+^; the ^1^H NMR spectrum (400 MHz, DMSO-*d*_6_) data were consistent with the literature [26] (Figures S1–S3). High-performance liquid chromatography (HPLC) analysis, ESI MS, and ^1^H NMR indicated that DOX-SS-Pyr was successfully synthesized. Then, DOX-SS-Pyr reacted with peptide Cys-^L^T7 or ^D^T7-Cys via a disulfide bond exchange reaction to generate the crude PDC, which was purified by semi-preparative HPLC. The HPLC purity of the purified ^L^T7-SS-DOX conjugate (3.42 mg, 31.5% yield) was 97.3%; ESI MS (*m*/*z*) [C_74_H_96_N_16_O_22_S_2_]: calculated, 1624.6; found, 813.3 [M + 2H]^2+^ (Figures S4 and S5). The HPLC purity of the purified ^D^T7-SS-DOX conjugate (3.15 mg, 43.9% yield) was 97.4%; ESI MS (*m*/*z*) [C_74_H_96_N_16_O_22_S_2_]: calculated, 1624.6; found, 813.4 [M + 2H]^2+^ (Figures S6 and S7).

### 2.2. Serum Stability and Drug Release of PDCs

To verify whether the characteristics of ^D^T7-SS-DOX constructed with ^D^T7 as the ligand can be improved in comparison with those of ^L^T7-SS-DOX under different in vitro mimetic physiological conditions and tumor microenvironment, the stability and reduction-responsive drug release behavior were investigated by co-incubating the PDCs with mouse serum and different concentrations of glutathione (GSH), respectively.

#### 2.2.1. Serum Stability

**Figure 1:**
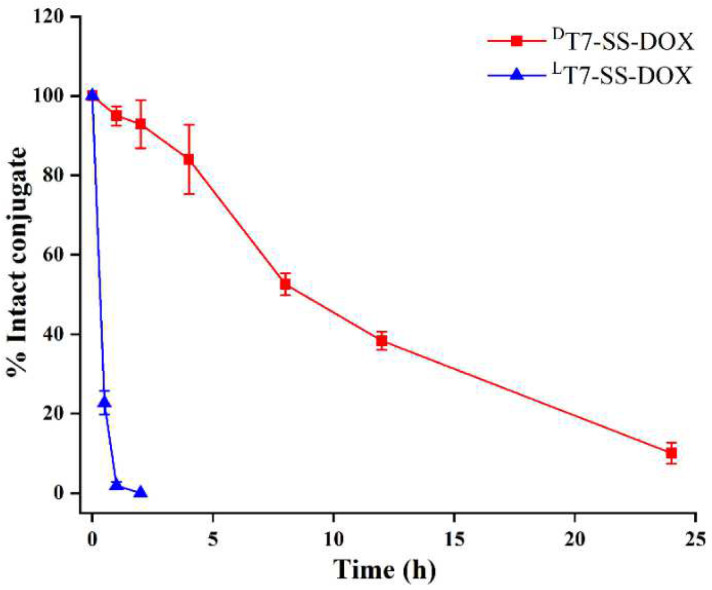
Stability analysis of PDCs in mouse serum.

With the extension of incubation time, both of the conjugates that remained intact in mouse serum decreased continuously (Figure 1). ^L^T7-SS-DOX was rapidly degraded within 0.5 h and was almost entirely degraded at 1 h. Whereas the degradation of ^D^T7-SS-DOX (*t*_1/2_ = 8.58 ± 0.85 h) was significantly slower than that of ^L^T7-SS-DOX (*t*_1/2_ = 0.37 ± 0.02 h) (*p* < 0.001). Namely, ^D^T7-SS-DOX was much more stable in mouse serum than ^L^T7-SS-DOX, indicating that the serum stability of the PDC constructed with ^D^T7 as the ligand can be prominently elevated.

#### 2.2.2. Reduction-Triggered Drug Release

**Figure 2:**
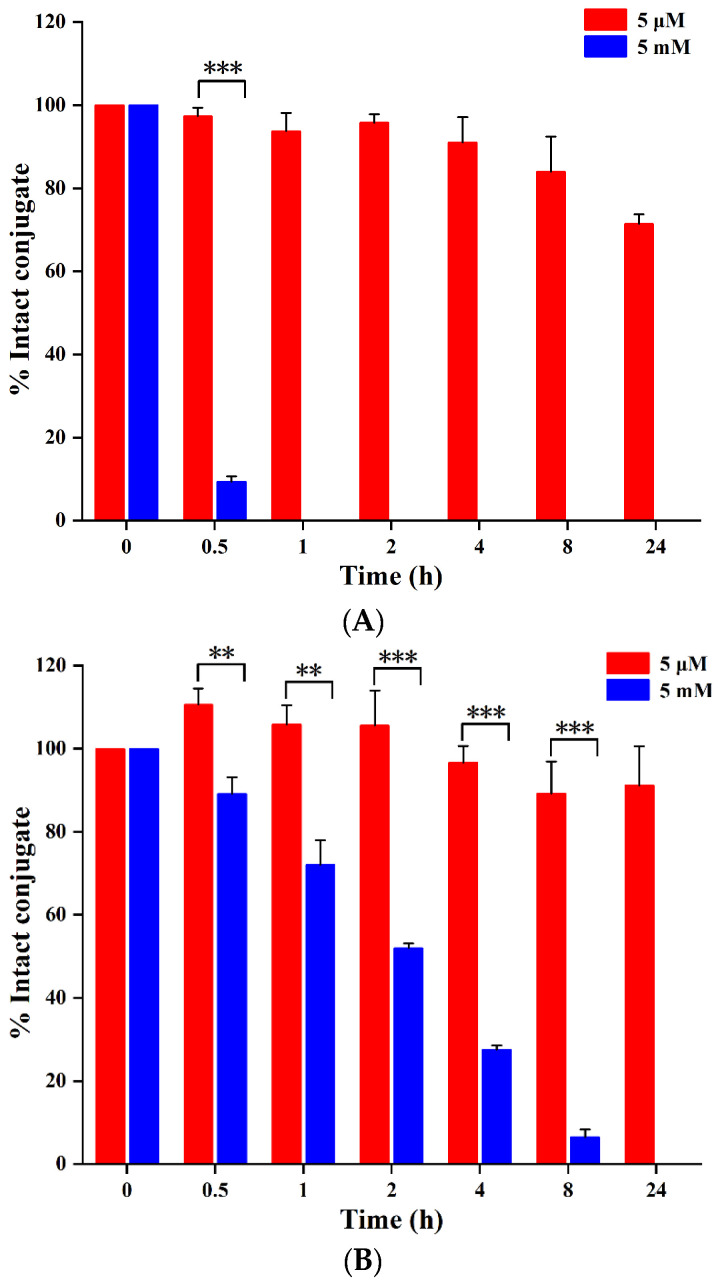
Reduction-triggered degradation of ^L^T7-SS-DOX (**A**) and ^D^T7-SS-DOX (**B**) by GSH. Data were expressed as mean ± SD (*n* = 3), ** *p* < 0.01 and *** *p* < 0.001.

As shown in Figure 2A, ^L^T7-SS-DOX was completely degraded in the presence of 5 mM GSH within 1 h. In contrast, the degradation of this conjugate in 5 μM GSH was much slower because 93.7% of the conjugate remained undegraded at 1 h, and 71.4% of the intact conjugate was detected when incubated for 24 h. As for ^D^T7-SS-DOX (Figure 2B), when incubated in 5 mM GSH, the conjugate that remained intact gradually decreased within 24 h. Overall, 72.1% of ^D^T7-SS-DOX remained undegraded at 1 h, and no intact conjugate was detected at 24 h. Nevertheless, this conjugate was more stable in the presence of 5 μM GSH because 91.2% of the conjugate still remained intact when incubated for 24 h.

It is noteworthy that the degradations of ^D^T7-SS-DOX in both 5 mM and 5 μM GSH were slower than that of ^L^T7-SS-DOX. In 5 mM GSH mimicking the tumor cell reductive microenvironment (2–10 mM GSH) [27], the ^D^T7-SS-DOX conjugate showed 50% degradation in approximately 2 h (*t*_1/2_ = 1.93 ± 0.13 h), which was almost 8 times longer than that of ^L^T7-SS-DOX (*t*_1/2_ = 0.26 ± 0.01 h), demonstrating that ^D^T7-SS-DOX had a sustained drug release manner which might help to exert the antitumor effect for a longer time. When incubated in 5 μM GSH, ^D^T7-SS-DOX was more stable than ^L^T7-SS-DOX during the incubation period of 24 h (Figure 2A,B), which might result in higher stability, less drug release, and toxicity of ^D^T7-SS-DOX in normal physiological condition.

### 2.3. Confocal Microscopy Analysis of Cellular Uptake

TfR overexpressed human A549, HepG2, U87 tumor cell lines [22,28], and TfR low-expressed human LO2 normal liver cell line [29] were used to evaluate the in vitro tumor-targeted cellular uptake of the conjugates by using laser confocal scanning microscopy.

**Figure 3:**
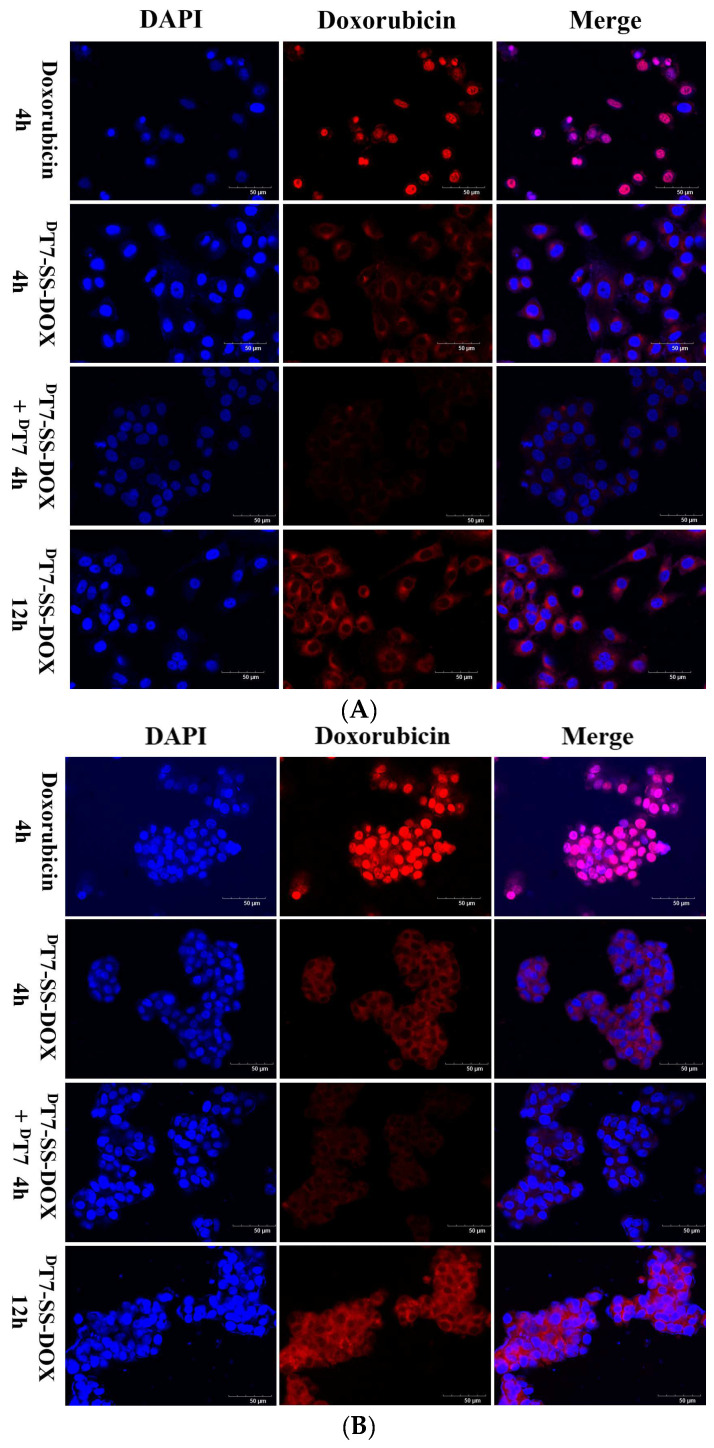

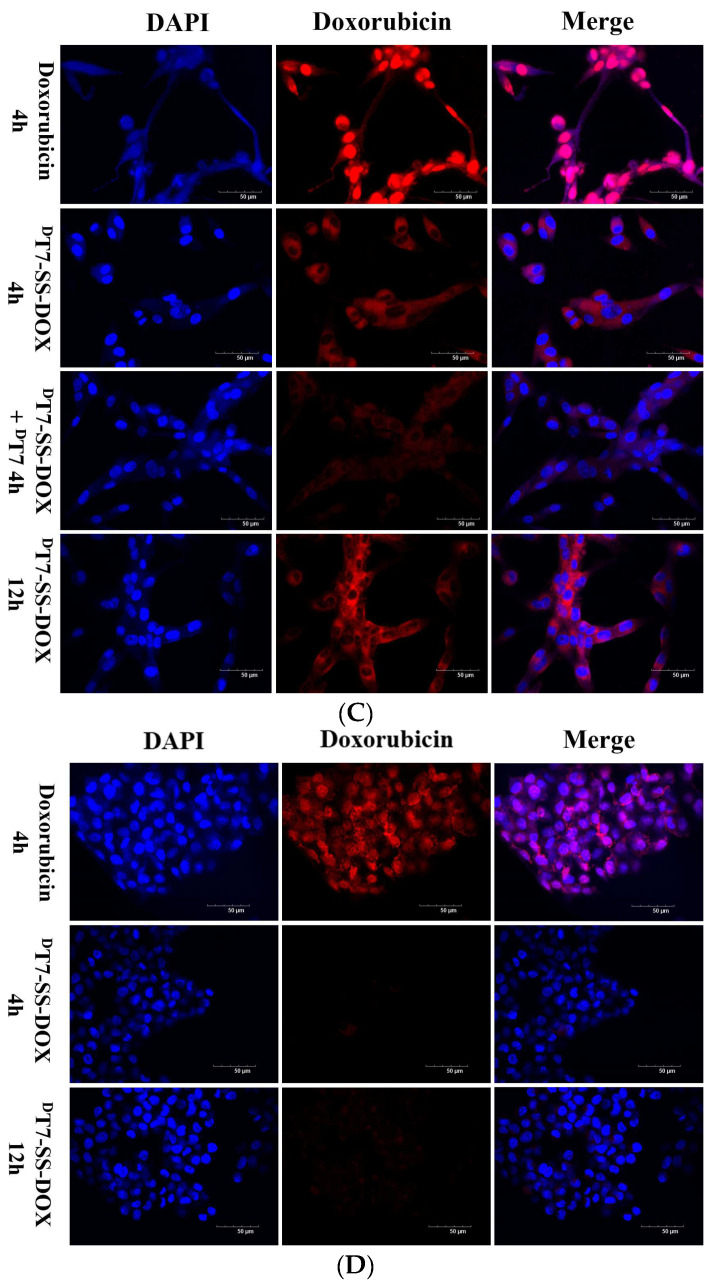
Confocal microscopy images of free DOX (10 µM, 4 h) and ^D^T7-SS-DOX (equivalent DOX concentration of 10 µM, 4 and 12 h) in A549 (**A**), HepG2 (**B**), U87 (**C**), and LO2 (**D**) cells. For the TfR competitive inhibition assay, cells were pre-incubated with the TfR affinity peptide (100 μM) for 8 h and then treated with ^D^T7-SS-DOX for 4 h. Blue represents the nuclei stained with DAPI, and red represents the fluorescence of DOX. Scale bars: 50 µm.

**Figure 4:**
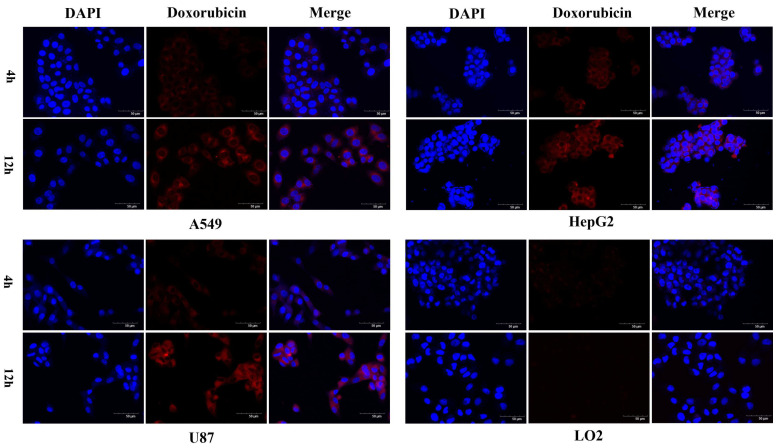
Confocal microscopy images of ^L^T7-SS-DOX (equivalent DOX concentration of 10 µM, 4 and 12 h) in A549, HepG2, U87, and LO2 cells. Blue represents the nuclei stained with DAPI, and red represents the fluorescence of DOX. Scale bars: 50 µm.

The cell nuclei stained with DAPI were shown as blue fluorescence, and DOX was detected with red fluorescence. As exhibited in Figure 3A–D, the red fluorescence located in the cell nuclei of A549, HepG2, U87, and LO2 cells after treatment with free DOX for 4 h, indicating that free DOX could enter into both tumor and normal cells due to its poor selectivity to tumor cells. As for cells treated with the conjugates for 4 and 12 h (Figure 3 and Figure 4), the red fluorescence was mainly distributed in the cytoplasm of A549, HepG2, and U87 tumor cells but can barely be found in LO2 cells, indicating that the conjugates can selectively enter into tumor cells, and free DOX was not released from the conjugates. Namely, the results demonstrated that the selectivity of DOX to TfR overexpressed tumor cells was effectively improved by conjugating with the TfR affinity peptides. Moreover, the cellular uptake pathway of the conjugates might be different from that of free DOX.

In a previous study, it was found that ^L^T7-, ^D^T7-, and Tf-modified liposomes (^L^T7-LIP, ^D^T7-LIP, and Tf-LIP) could effectively enter into HepG2 cells without pre-incubation of the corresponding TfR-targeting ligands, indicating that the three ligands maintained binding affinity to TfR after conjugation with liposomes. However, the pre-incubation of HepG2 cells with the TfR affinity peptide (^L^T7 or ^D^T7) significantly reduced the cellular uptake of both ^L^T7-LIP and ^D^T7-LIP but did not decrease the uptake of Tf-LIP. These results demonstrated that the binding site of ^D^T7 on the TfR might be the same as that of ^L^T7 but was different from that of Tf [22]. Based on these findings, we speculated that the cellular uptake of our PDCs might also be related to the binding of the peptide ligands to TfR. To investigate the cellular uptake pathway of the conjugates, a TfR competitive inhibition assay was utilized. The results revealed that the fluorescence intensity of ^D^T7-SS-DOX in A549, HepG2, and U87 tumor cells pre-treated with the TfR affinity peptide was lower than that of the corresponding type of tumor cells without pre-incubation of the TfR affinity peptide (Figure 3A–C), indicating that pre-occupation of the binding site of TfR overexpressed on the surface of tumor cells with the TfR affinity peptide clearly reduced the uptake of ^D^T7-SS-DOX by cells, suggesting that the conjugate might enter into cells via a TfR-mediated endocytosis pathway.

When incubated with drugs for 4 h, the fluorescence intensity in the same kind of tumor cells ranking from the highest to the lowest was free DOX, ^D^T7-SS-DOX, and ^L^T7-SS-DOX (Figure 3A–C and Figure 4). This phenomenon is probably due to the fast passive diffusion of free DOX into cells without a drug release process, whereas the entry of the conjugates into cells via TfR-mediated endocytosis was a relatively slow process. Meanwhile, the binding affinity of ^D^T7 to TfR is higher than that of ^L^T7, so ^D^T7-SS-DOX could bring more drugs into tumor cells via the TfR-mediated endocytosis pathway during the same incubation period. In addition, the fluorescence intensity of both ^L^T7-SS-DOX and ^D^T7-SS-DOX in tumor cells increased with the prolongation of incubation time (Figure 3A–C and Figure 4, 4 h versus 12 h), demonstrating that the TfR-mediated endocytosis of PDCs and the subsequent cleavage of the disulfide bond by GSH to generate DOX-SH (Scheme 1) was a gradually cumulative process which was time-dependent.

### 2.4. In Vitro Cytotoxicity

**Figure 5:**
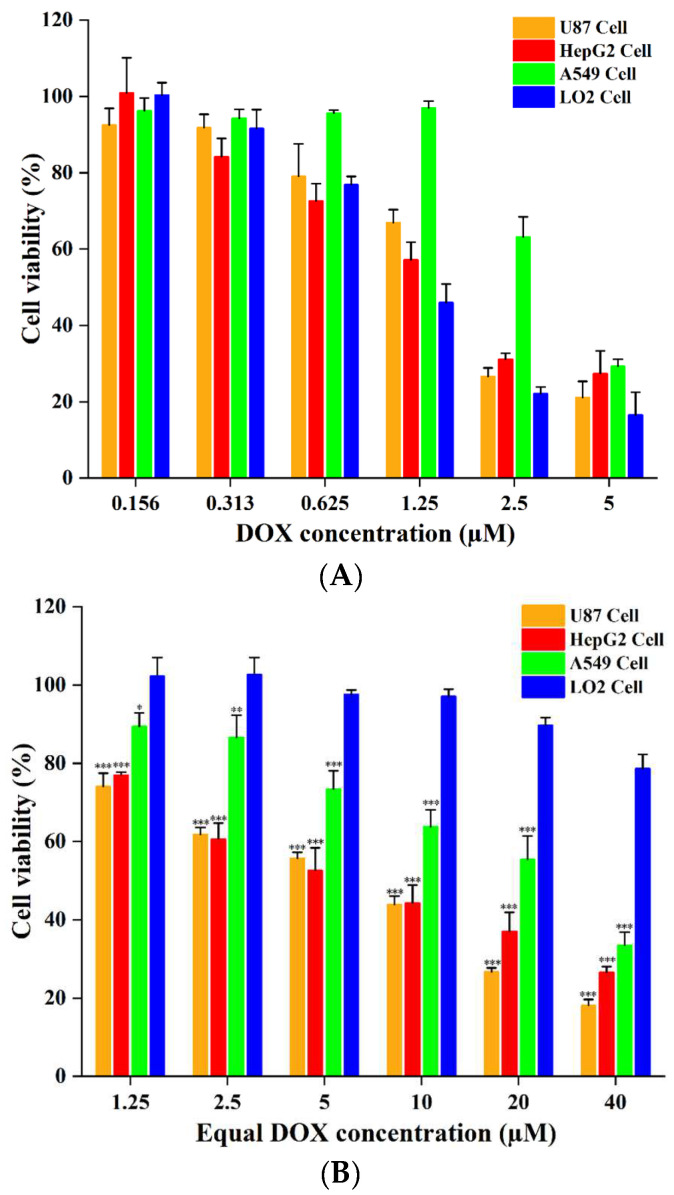
In vitro cytotoxicity of free DOX (**A**) and ^D^T7-SS-DOX (**B**) against cells. Data were presented as mean ± SD (*n* = 3), * *p* < 0.05; ** *p* < 0.01 and *** *p* < 0.001 versus LO2 cells group.

The in vitro cytotoxicities of free DOX, ^L^T7-SS-DOX, and ^D^T7-SS-DOX against U87, HepG2, A549, and LO2 cells were investigated by CCK-8 assay. As revealed in Figure 5 and Figure S8, the cell viability of each kind of cell line after incubation with free DOX, ^L^T7-SS-DOX, and ^D^T7-SS-DOX decreased in a dose-dependent manner. The antiproliferative activity of drugs against tumor cells ranking from the highest to the lowest was free DOX, ^D^T7-SS-DOX, and ^L^T7-SS-DOX, but the toxicities of the two conjugates to LO2 normal cells were much lower than that of free DOX, which was consistent with the aforementioned results of cellular uptake. DOX showed strong in vitro cytotoxicity against all four kinds of cells without tumor selectivity (Figure 5A), with IC_50_ values of 1.65 ± 0.20 µM (U87), 1.58 ± 0.19 µM (HepG2), 3.55 ± 0.21 µM (A549), and 1.29 ± 0.18 µM (LO2), respectively (Table 1). ^D^T7-SS-DOX exhibited good in vitro antiproliferative activity against the three tumor cell lines (Figure 5B), with IC_50_ values of 5.70 ± 0.22 µM (U87), 7.01 ± 1.64 µM (HepG2), and 20.61 ± 4.81 µM (A549), respectively (Table 1). The proliferation inhibitory activity of ^L^T7-SS-DOX was the weakest among the three drugs because the cell viabilities of U87, HepG2, and A549 cells after incubation with ^L^T7-SS-DOX (equal DOX concentration of 20 µM) for 48 h were 95.1%, 73.1%, and 83.2%, respectively. Even at an equal DOX concentration of 40 µM, the cell viability of the three types of tumor cells after exposure to this conjugate for 48 h were 41.0%, 61.5%, and 67.2%, respectively (Figure S8). Because of the inconspicuous antiproliferative activity of ^L^T7-SS-DOX at an equal DOX concentration of 20 µM, drugs below this concentration were not used.

**Figure 6:**
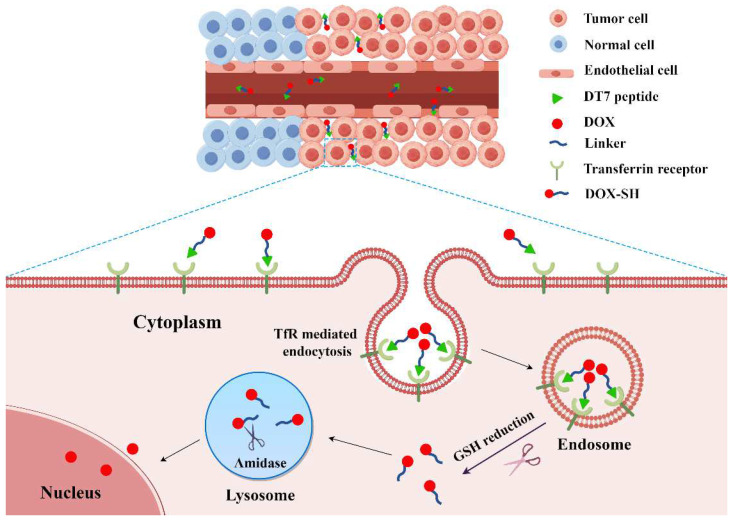
Schematic illustration of the stability in blood circulation, cellular uptake pathway, and drug release behavior of ^D^T7-SS-DOX in TfR overexpressed tumor cells (Created by Figdraw).

The reason for the stronger antitumor activity of free DOX is possible because this drug can quickly enter into tumor cells via passive diffusion and immediately kill tumor cells in vitro. However, it is supposed that PDCs constructed by forming an amide bond via the amino of DOX may not exert cell proliferation inhibitory activity until free DOX is released. One of the PDCs like this, named cRGD-SS-DOX, released DOX-SH rather than free DOX after co-incubation with B16 mouse melanoma cells for 3 h. Furthermore, the in vitro cytotoxicity of cRGD-SS-DOX against B16 cells was found to be 20.6 times lower than that of free DOX [30]. Similarly, after taken up by tumor cells, we speculated that the peptide–doxorubicin conjugates in our study can exhibit antiproliferative activity only if the disulfide bond is cleaved by GSH to generate DOX-SH, and the subsequent cleavage of the amide bond of DOX-SH by amidases present in lysosomes to release free drug finally occurs, as illustrated in Figure 6. Nevertheless, cellular uptake results of the above PDCs showed that the red fluorescence was still mainly located in the cytoplasm of tumor cells even at an incubation time of 12 h, indicating that the release of free DOX was a slow and time-dependent process, which made the antiproliferative activity of PDCs weaker than that of free DOX. In addition, the stronger antiproliferative activity of ^D^T7-SS-DOX in comparison with that of ^L^T7-SS-DOX was probably related to the higher affinity of ^D^T7-SS-DOX to TfR as well as its sustained drug release behavior in tumor cells.

It is worth mentioning that although the in vitro antiproliferative activity of ^D^T7-SS-DOX against U87, HepG2, and A549 tumor cells was 3.4, 4.4, and 5.8 times, respectively, less potent than that of free DOX, its toxicity to LO2 normal cells (IC_50_ value > 100 µM) was significantly reduced compared with that of free DOX (1.29 ± 0.18 µM) (Table 1), indicating that this conjugate had an excellent selectivity to TfR overexpressed tumor cells due to the targeting effect of the ^D^T7 peptide ligand, which is of significant importance considering the clinical application of DOX is severely restricted by its poor tumorous selectivity. Moreover, given that insufficient stability is the major drawback of PDCs constructed with L-peptide ligands, the remarkedly enhanced serum stability of the ^D^T7-SS-DOX conjugate is crucial to ensure its integrity in blood circulation and maintain the targeting effect of the peptide ligand on TfR after reaching the tumor site, which might facilitate the accumulation of drugs in target tumor cells, as depicted in Figure 6.

## 3. Materials and Methods

### 3.1. Materials

Doxorubicin hydrochloride (DOX·HCl, 98%) was purchased from Meilun Biotechnology Co., Ltd. (Dalian, China); Cys-^L^T7 (97.9%) was synthesized by our group as previously described [31]; ^D^T7-Cys (99.2%) was provided by Chinapeptides BioTech Co., Ltd. (Shanghai, China); SPDP (96%) was obtained from J&K Scientific Co., Ltd. (Beijing, China); GSH (98%) was purchased from Sigma-Aldrich (Wuxi, China); CCK-8 was obtained from APExBIO Technology (Shanghai, China); 4′, 6-diamidino-2-phenylindole (DAPI) solution was purchased from Solarbio Life Sciences Co., Ltd. (Beijing, China); mouse serum was provided by Sbjbio Tech Co., Ltd. (Nanjing, China). All other chemicals were of analytical or chromatographic grade.

Human HepG2 hepatocellular carcinoma cells and human U87 glioblastoma cells were purchased from Procell Life Science & Technology Co., Ltd. (Wuhan, China), human A549 lung carcinoma cells were obtained from the School of Basic Medical Sciences, Chengde Medical University, human LO2 normal hepatic cells were purchased from Jennio Biotech Co., Ltd. (Guangzhou, China). The cells were cultured in DMEM (HepG2), MEM containing 1% non-essential amino acids (U87), and RPMI-1640 (A549 and LO2), supplemented with 10% fetal bovine serum (FBS) and 1% penicillin/streptomycin at 37 °C in a humidified atmosphere with 5% CO_2_.

### 3.2. Synthesis of Peptide–DOX Conjugates

#### 3.2.1. Synthesis of DOX-SS-Pyr

DOX-SS-Pyr was synthesized according to the published procedure [32] with minor modifications. Briefly, *N*, *N*-Diisopropylethylamine (DIPEA, 19 μL, 109.1 μmol) was added dropwise to a stirred solution of DOX·HCl (30.6 mg, 52.8 μmol) and SPDP (20.1 mg, 64.3 μmol) in 2.2 mL of anhydrous *N*, *N*-Dimethylformamide (DMF), and the mixture was stirred at room temperature for 2 h. The process of the reaction was monitored by analytical HPLC. Then, the reaction solution was treated with deionized water, and the resulting precipitate was collected by centrifugation and dried under vacuum to give DOX-SS-Pyr as a red solid, which was characterized by HPLC, ESI MS, and ^1^H NMR. HPLC analyses in this study were all performed on a ZORBAX Eclipse XDB-C18 reversed-phase column (250 mm × 4.6 mm, 5 μm; Agilent, Santa Clara, CA, USA) connected to an Agilent 1200 HPLC system running with a linear gradient of 5–95% acetonitrile/water with 0.1% trifluoroacetic acid at 1 mL/min in 20 min. The monitoring wavelength was 254 nm.

#### 3.2.2. Synthesis of Peptide–DOX Conjugates

The PDC was synthesized via a disulfide bond exchange reaction between the free sulfydryl in the structure of peptide Cys-^L^T7 or ^D^T7-Cys and the 2-pyridyldithio group in the DOX-SS-Pyr. Briefly, DOX-SS-Pyr (5.0 mg, 6.8 μmol) and Cys-^L^T7 peptide (6.5 mg, 6.5 μmol) were dissolved in 0.7 mL of anhydrous DMF, and the mixture was stirred under a nitrogenous atmosphere at room temperature for 1 h. For the synthesis of ^D^T7-SS-DOX, DOX-SS-Pyr (3.9 mg, 5.3 μmol), and ^D^T7-Cys peptide (4.3 mg, 4.3 μmol) were dissolved in 0.5 mL of anhydrous DMF, and the mixture was stirred under a nitrogenous atmosphere at room temperature for 6 h. The process of the reaction was monitored by analytical HPLC. Upon completion of the reaction, the resulting ^L^T7-SS-DOX or ^D^T7-SS-DOX conjugate was purified by semi-preparative HPLC conducted on an Ultimate XB-C18 reversed-phase column (250 mm × 10 mm, 5 μm; Welch, Shanghai, China). The mobile phases consisted of solvent A (0.1% formic acid in water) and solvent B (0.1% formic acid in acetonitrile). An isocratic elution of 21% eluent B over 20 min and a linear gradient of 5–70% eluent B over 70 min were applied for ^L^T7-SS-DOX and ^D^T7-SS-DOX purification, respectively. The flow rate was 2 mL/min, and peaks were detected at 215 nm. Fractions were collected, evaporated, and lyophilized as a red powder, which was characterized by analytical HPLC and ESI MS.

### 3.3. In Vitro Stability of Conjugates

The in vitro stability experiment of the conjugates was conducted by co-incubation with mouse serum, as previously described [33], with minor modifications. An 80 μL of ^L^T7-SS-DOX or ^D^T7-SS-DOX stock solution (1 mM) was diluted by adding ultra-pure water (520 μL) and mouse serum (200 μL). The mixture was incubated at 37 °C, aliquots (100 μL) were taken at scheduled time intervals, and then methanol (300 μL) was added to terminate the incubation. After centrifugation at 10,000 rpm for 10 min, the intact PDC that remained in the supernatant was analyzed by HPLC.

### 3.4. In Vitro Drug Release of Conjugates

A stock solution of ^L^T7-SS-DOX or ^D^T7-SS-DOX was diluted by adding 5 mM or 5 μM GSH dissolved in acetonitrile-PBS (3:7, *v*/*v*) to a final volume of 800 μL with 100 μM conjugate, and the mixture was incubated at 37 °C. Aliquots (100 μL) were taken from the incubation solution at scheduled time intervals and diluted with acetonitrile–PBS (3:7, *v*/*v*, 200 μL). After filtration, the intact conjugate that remained in the sample was analyzed by HPLC.

### 3.5. Confocal Microscopy Studies

A549, HepG2, U87, or LO2 cells were seeded on glass coverslips (5 × 10^4^ cells/well) in 24-well cell culture plates and incubated at 37 °C for 24 h. Then, cells were treated with the peptide–DOX conjugates (equivalent DOX concentration of 10 μM) for 4 and 12 h, respectively. Cells treated with free DOX (10 μM) for 4 h were used as the positive control. After the drugs in each well were discarded, cells were washed three times with PBS and fixed with 4% paraformaldehyde for 12 h. Cell nuclei were stained with DAPI for 8 min. The fluorescent images of cells were observed by using a confocal laser scanning microscope (Olympas FV3000).

For the TfR competitive inhibition assay, A549, HepG2, or U87 cells seeded on glass coverslips in 24-well cell culture plates were incubated at 37 °C overnight and then pre-treated with serum-free cultural medium containing the TfR affinity peptide (100 μM) at 37 °C for 8 h. After removal of the peptide solution, cells were washed three times with PBS and incubated with the ^D^T7-SS-DOX conjugate (equivalent DOX concentration of 10 μM) for 4 h. Cell fixation, staining, and cellular uptake analysis by confocal microscopy were conducted as aforementioned methods.

### 3.6. In Vitro Cytotoxicity Assay

A549, HepG2, U87, and LO2 cells were each seeded in 96-well cell culture plates at a density of 6 × 10^3^ cells per well and incubated at 37 °C for 24 h. Then, cells were treated with serial concentrations of free DOX or the peptide–DOX conjugates for 48 h. After incubation, CCK-8 (10 μL) was added to each well and incubated for an additional 2 h. The absorbance of each well was measured at 450 nm with a reference wavelength of 630 nm by using a Multiskan GO microplate reader (Thermo Fisher Scientific, Waltham, MA, USA). Data for each cell line treated with free DOX or PDCs were obtained from three independent experiments.

### 3.7. Statistical Analysis

Data were presented as the mean ± SD. All statistical analyses were performed using SPSS 26. The comparison of parameters among the two groups was made by the unpaired Student’s *t*-test. Group differences were determined by one-way analysis of variance (ANOVA) followed by Fisher’s LSD post hoc. The differences were considered to be significant at *p* < 0.05.

## 4. Conclusions

In this present study, two new TfR affinity peptide–doxorubicin conjugates were designed and synthesized. Compared with ^L^T7-SS-DOX, the ^D^T7-SS-DOX conjugate had the advantages of much higher in vitro serum stability, more sustained reduction-responsive drug release behavior, and stronger tumor cell proliferation inhibitory activity, indicating that ^D^T7 can be used as an excellent peptide ligand for constructing stable TfR targeted PDCs. In view of that the in vitro antiproliferative activity of ^D^T7-SS-DOX against tumor cells is less potent than that of free DOX, PDCs with an acid-triggered cleavable linkage such as hydrazone or ester bond between ^D^T7 and DOX need to be investigated in the future so as to release free DOX more efficiently within tumor cells and further improve the antitumor efficacy.

## Figures and Tables

**Table 1:** IC_50_ of ^D^T7-SS-DOX and free DOX on the four cell lines. Data were presented as mean ± SD (*n* = 3).

Compound	IC_50_ (µM)			
	U87	HepG2	A549	LO2
^D^T7-SS-DOX	5.70 ± 0.22	7.01 ± 1.64	20.61 ± 4.81	>100
DOX	1.65 ± 0.20	1.58 ± 0.19	3.55 ± 0.21	1.29 ± 0.18

## Data Availability

Data are contained within the article and Supplementary Materials.

## Supplementary Materials

The following supporting information can be downloaded at https://www.mdpi.com/article/10.3390/molecules29081758/s1. Figures S1–S3: HPLC chromatogram, ESI MS, and ^1^H NMR spectrum of DOX-SS-Pyr; Figures S4–S5: HPLC chromatogram and ESI MS of the ^L^T7-SS-DOX conjugate; Figures S6–S7: HPLC chromatogram and ESI MS of the ^D^T7-SS-DOX conjugate; Figure S8: In vitro cytotoxicity of ^L^T7-SS-DOX against cells.
