# Supplementary material for: New Transferrin Receptor-Targeted Peptide–Doxorubicin Conjugates: Synthesis and In Vitro Antitumor Activity
Source: Molecules. 2024 Apr 12;29(8):1758. doi: 10.3390/molecules29081758 (PMC11052316; doi:10.3390/molecules29081758)
Supplement: Supplementary file 1 [file molecules-29-01758-s001.zip › molecules-2933510-supplementary.pdf]

Supplementary material

New Transferrin Receptor-Targeted Peptide–Doxorubicin Conjugates: Synthesis and In Vitro Antitumor Activity

Jiale Yu [1], Xiaoxia Mao [1], Xue Yang [2], Guiqin Zhao [1] and Songtao Li [1,*]

1 Hebei Province Key Laboratory of Research and Development of Traditional Chinese Medicine, Institute of Chinese Mateia Medica, Chengde Medical University, Chengde 067000, China; 15102526322@163.com (J.Y.); mao0505@163.com (X.M.); zhaoguiqin1971@sina.com (G.Z.)
2 School of Basic Medical Sciences, Chengde Medical University, Chengde 067000, China; xyang_cdmu@163.com
* Correspondence: songtao-li@hotmail.com

Contents

Characterization of DOX-SS-Pyr and the PDCs……………………………………S1-S7

In vitro cytotoxicity of the ^{L}T7-SS-DOX conjugate…………………………………S8

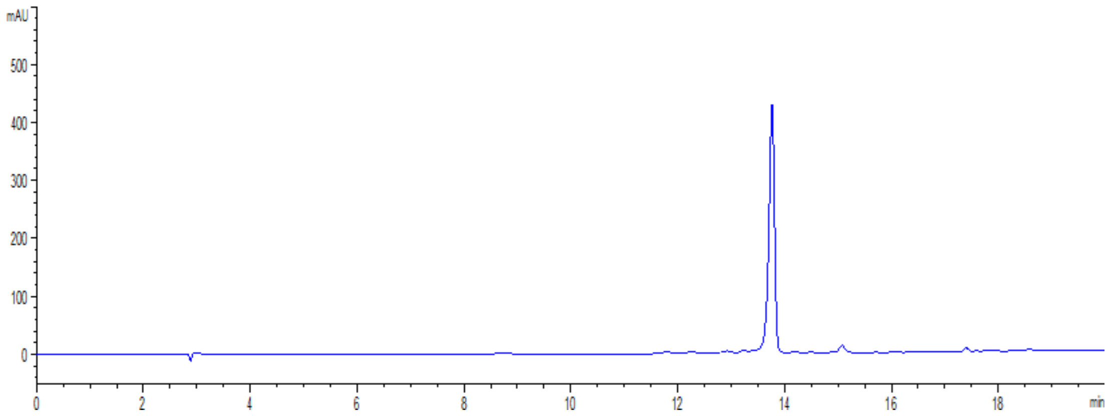

Figure S1. HPLC chromatogram of DOX-SS-Pyr

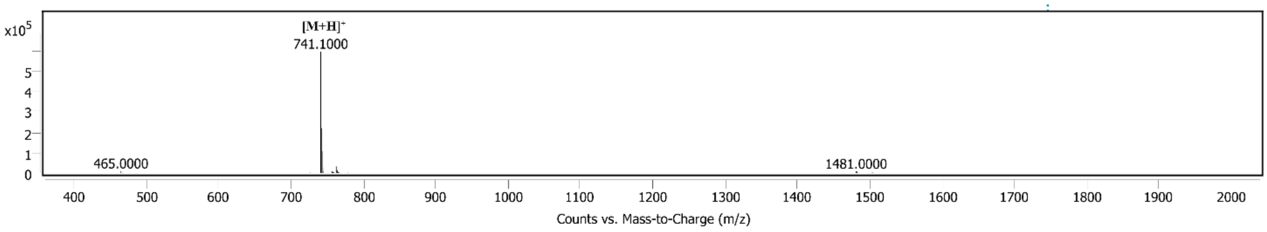

Figure S2. ESI MS of DOX-SS-Pyr, molecular weight for $[C_{35}H_{36}N_2O_{12}S_2]$: calculated, 740.2; found, 741.1 $[M + H]^+$

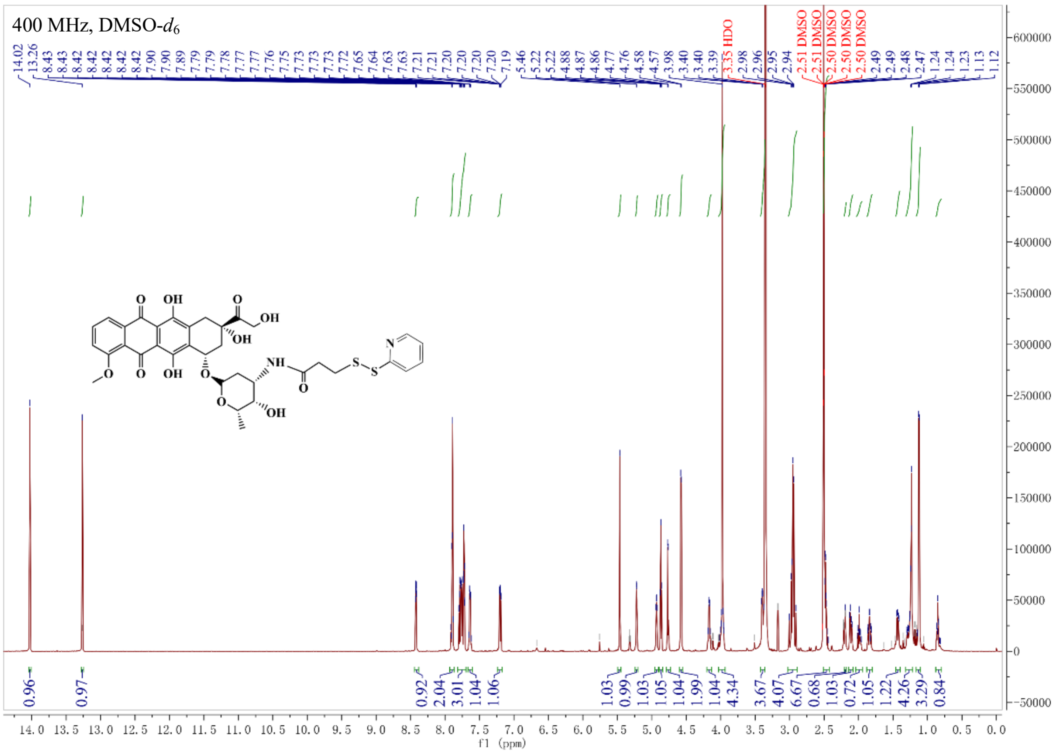

Figure S3. ^1H NMR spectrum of DOX-SS-Pyr

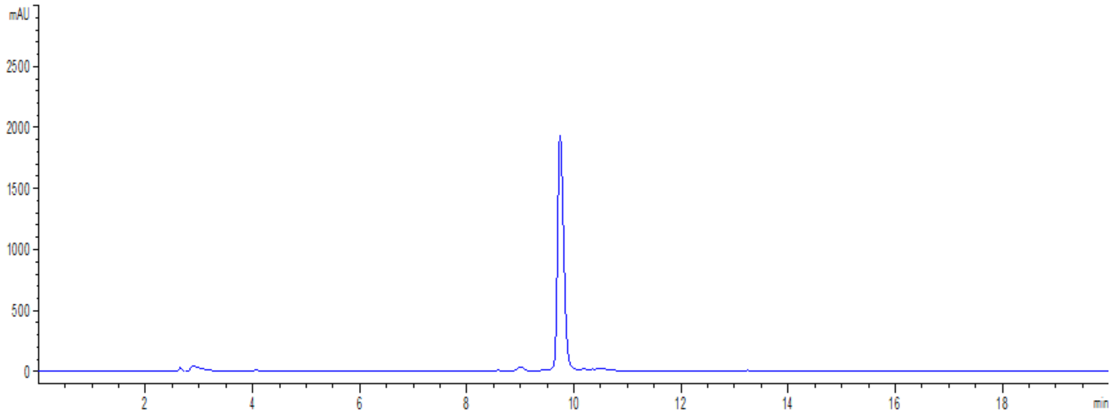

Figure S4. HPLC chromatogram of the ^{L}T7-SS-DOX conjugate

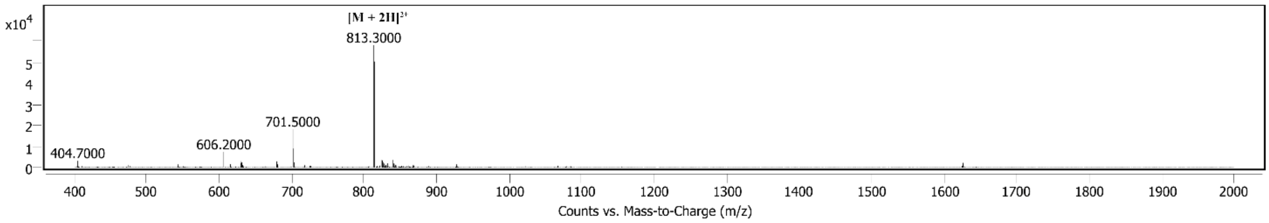

Figure S5. ESI MS of the ^{L}T7-SS-DOX conjugate, molecular weight for

[$C_{74}H_{96}N_{16}O_{22}S_2$]: calculated, 1624.6; found, 813.3 $[M + 2H]^{2+}$

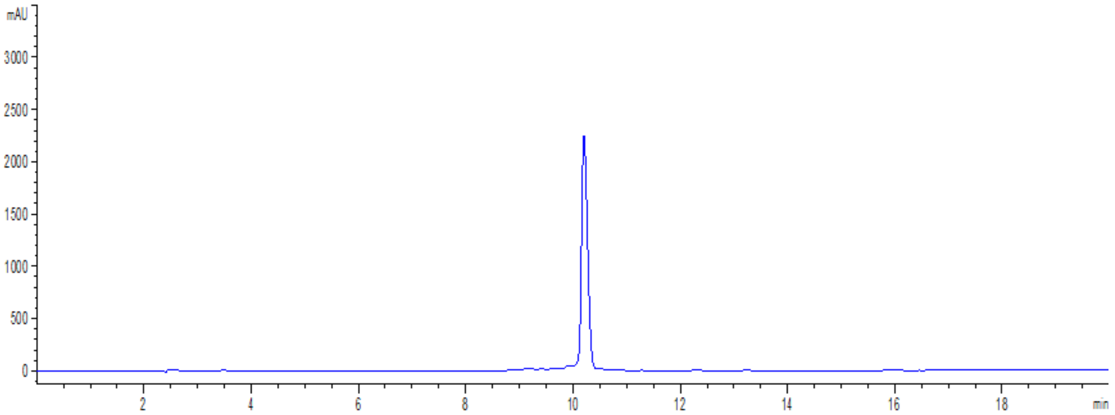

Figure S6. HPLC chromatogram of the ^{D}T7-SS-DOX conjugate

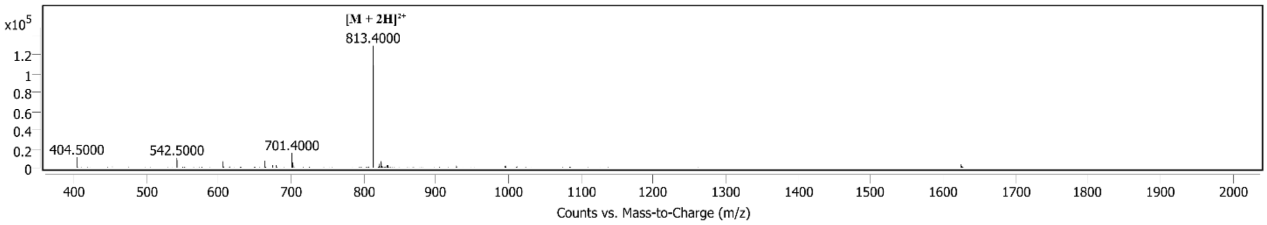

Figure S7. ESI MS of the ^{D}T7-SS-DOX conjugate, molecular weight for

[$C_{74}H_{96}N_{16}O_{22}S_2$]: calculated, 1624.6; found, 813.4 $[M + 2H]^{2+}$

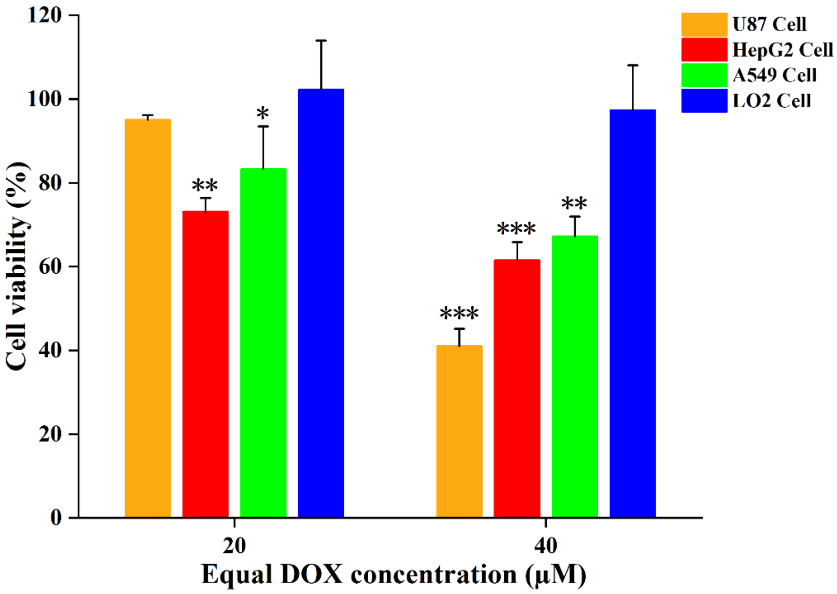

Figure S8. *In vitro* cytotoxicity of ^{L}T7-SS-DOX against cells. Data were presented as mean ± SD (n = 3), *p < 0.05; **p < 0.01 and ***p < 0.001 versus LO2 cells group.
